# Gastric Ulcers

**Published:** 1915-06

**Authors:** 


**Gastric Ulcers.** Frank Smithies, Chicago, Interstate Med. Jour., Mch. 1915. In an analysis of 500 cases operated upon, the following results were obtained: (In a larger series of 1725 cases, 1225 were duodenal and 500 gastric, 2.45:1). A plurality of cases were between 40 and 50 and more than three-fourths between 30 and 60. There were 315 males and 185 females. Nationality, occupation, habits, dietetic errors were not significant, though the author thinks the occurrence of 31.4 in American farmers to be so. A history of typhoid was present in 17%, of syphilis in 2.4% and various other infections were noted, there having apparently been a tendency to development of ulcer or exacerbation of symptoms immediately following an infectious process. In 36% the appendix was diseased or had been so, in 14% cholecystitis or cholelithiasis was or had been present. Thus, 50% of ulcers were at some time complicated with or followed these two processes. Macroscopic haemorrhage had been noted in 36.4% and haemathemesis in 80% of these. Blood was found macroscopically or by chemic test in 39% of stomach contents examined but, in some instances was apparently due to intubation. In retention cases, free HCl averaged 56.4 degrees, total acidity 74.2; in other cases, 40.5 and 52.4. He speaks deprecatingly of the string test, blood stains being found in only 7 instances in 318 cases of ulcer. He emphasizes the intermittency of bleeding and the exaggeration of blood tests by failure to exclude meat—31% of cases showing blood at a given time after proper diet, the majority without this precaution. (Note: The majority of faeces, examined as a routine without limitation to any group of cases, react negatively to blood by course applications of the various oxidase tests, guaiac, phenolphthalin, aloin, benzidin, etc., even in the presence of meat. Thus, while meat may cause a blood test adventitiously, it does not often do so if too delicate tests are excluded. The reason for this ought to be carefully worked out. Possibly, it will not only afford means of checking true bleeding but a clue as to digestive and absorptive activities. Ed.). X-ray methods are considered as of the same relative worth as differential blood counts in anaemia, as valuable but not conclusive, especially not in uncomplicated cases. The importance of fluoroscopic examination, in addition to plate tests and of personal inspection of X-ray plates and screens by the attendant, are emphasized. He speaks guardedly and without numeric statements, as to prognosis. Various statistics of somewhat indirect nature are given as to the tendency of gastric ulcer to develop cancer, there being a real danger of such degeneration but not so marked as extremists have stated.

